# Supplementary material for: Empirical methods for controlling false positives and estimating confidence in ChIP-Seq peaks
Source: BMC Bioinformatics. 2008 Dec 5;9:523. doi: 10.1186/1471-2105-9-523 (PMC2628906; doi:10.1186/1471-2105-9-523)
Supplement: Additional File 1 — A variety of html documents from the USeq web site detailing the available applications, their best usage, output file type descriptions, command line menus, etc. [file 1471-2105-9-523-S1.zip › USeqUserGuides/JavaDoc.zip!/JavaDoc/bioroot/antibody/AntibodyPrep.html]

AntibodyPrep


|  |  |  |
| --- | --- | --- |
| **Overview** \| **Package** \| **Class** \| **Use** \| **Tree** \| **Deprecated** \| **Index** \| **Help** | |  |
| **PREV CLASS**   **NEXT CLASS** | **FRAMES**    **NO FRAMES**     **All Classes** |  |
| SUMMARY: NESTED \| FIELD \| CONSTR \| METHOD | DETAIL: FIELD \| CONSTR \| METHOD |  |


---


## bioroot.antibody Class AntibodyPrep

```
java.lang.Object
  bioroot.antibody.AntibodyPrep
```

---

``` public class AntibodyPrep extends java.lang.Object ```

**Author:**
:   nix
    This contains information related to a particular antibody bleed, hybridoma sup collection, or purification

---

**Constructor Summary**

`AntibodyPrep()`

`AntibodyPrep(int antibodyId,
java.sql.ResultSet results)`


| **Method Summary** | |
| --- | --- |
| `int` | `getAntibodyId()` |
| `java.lang.String` | `getDate()` |
| `java.lang.String` | `getFileName()` |
| `int` | `getId()` |
| `java.lang.String` | `getLastUser()` |
| `java.lang.String` | `getLocation()` |
| `java.lang.String` | `getName()` |
| `java.lang.String` | `getNotes()` |
| `java.lang.String` | `getOldFileName()` |
| `int` | `getOrganismId()` |
| `java.lang.String` | `getOrganismName(DBUtil bioRoot)` |
| `java.lang.String` | `getPurification()` |
| `java.lang.String` | `getRecipients()` |
| `java.lang.String` | `getRemainingAliquots()` |
| `UseDilution[]` | `getUseDilutions(DBUtil bioRoot)` |
| `java.lang.String` | `getUserComments()` |
| `boolean` | `saveNewUseDilutions(DBUtil bioRoot)` |
| `void` | `setAntibodyId(int antibodyId)` |
| `void` | `setDate(java.lang.String date)` |
| `void` | `setFileName(java.lang.String fileName)` |
| `void` | `setId(int id)` |
| `void` | `setLastUser(java.lang.String lastUser)` |
| `void` | `setLocation(java.lang.String location)` |
| `void` | `setName(java.lang.String name)` |
| `void` | `setNotes(java.lang.String notes)` |
| `void` | `setOldFileName(java.lang.String oldFileName)` |
| `void` | `setOrganismId(int i)` |
| `void` | `setOrganismName(java.lang.String organismName)` |
| `void` | `setPurification(java.lang.String purification)` |
| `void` | `setRecipients(java.lang.String recipients)` |
| `void` | `setRemainingAliquots(java.lang.String remainingAliquots)` |
| `void` | `setUseDilutions(UseDilution[] useDilutions)` |
| `void` | `setUserComments(java.lang.String userComments)` |
| `boolean` | `submitNew(DBUtil bioRoot)`             Makes a new AntibodyPrep table entry. |
| `boolean` | `updateCommonAccessFields(DBUtil bioRoot)`             Just updates fields that are accessible by all lab group memebers. |
| `boolean` | `updateOld(DBUtil bioRoot)`             Updates a Antibody prep entry |

**Methods inherited from class java.lang.Object**

`equals, getClass, hashCode, notify, notifyAll, toString, wait, wait, wait`

**Constructor Detail**

### AntibodyPrep

```
public AntibodyPrep(int antibodyId,
                    java.sql.ResultSet results)
```

---


### AntibodyPrep

```
public AntibodyPrep()
```


**Method Detail**

### submitNew

```
public boolean submitNew(DBUtil bioRoot)
```

:   Makes a new AntibodyPrep table entry.

---


### updateOld

```
public boolean updateOld(DBUtil bioRoot)
```

:   Updates a Antibody prep entry

---


### saveNewUseDilutions

```
public boolean saveNewUseDilutions(DBUtil bioRoot)
```

---


### getOrganismName

```
public java.lang.String getOrganismName(DBUtil bioRoot)
```

---


### getOrganismId

```
public int getOrganismId()
```

---


### setOrganismId

```
public void setOrganismId(int i)
```

---


### setOrganismName

```
public void setOrganismName(java.lang.String organismName)
```

---


### getUseDilutions

```
public UseDilution[] getUseDilutions(DBUtil bioRoot)
```

---


### updateCommonAccessFields

```
public boolean updateCommonAccessFields(DBUtil bioRoot)
```

:   Just updates fields that are accessible by all lab group memebers.

---


### getAntibodyId

```
public int getAntibodyId()
```

---


### setAntibodyId

```
public void setAntibodyId(int antibodyId)
```

---


### getDate

```
public java.lang.String getDate()
```

---


### setDate

```
public void setDate(java.lang.String date)
```

---


### getId

```
public int getId()
```

---


### setId

```
public void setId(int id)
```

---


### getLastUser

```
public java.lang.String getLastUser()
```

---


### setLastUser

```
public void setLastUser(java.lang.String lastUser)
```

---


### getNotes

```
public java.lang.String getNotes()
```

---


### setNotes

```
public void setNotes(java.lang.String notes)
```

---


### getPurification

```
public java.lang.String getPurification()
```

---


### setPurification

```
public void setPurification(java.lang.String purification)
```

---


### getRecipients

```
public java.lang.String getRecipients()
```

---


### setRecipients

```
public void setRecipients(java.lang.String recipients)
```

---


### getRemainingAliquots

```
public java.lang.String getRemainingAliquots()
```

---


### setRemainingAliquots

```
public void setRemainingAliquots(java.lang.String remainingAliquots)
```

---


### setUseDilutions

```
public void setUseDilutions(UseDilution[] useDilutions)
```

---


### getUserComments

```
public java.lang.String getUserComments()
```

---


### setUserComments

```
public void setUserComments(java.lang.String userComments)
```

---


### getFileName

```
public java.lang.String getFileName()
```

---


### setFileName

```
public void setFileName(java.lang.String fileName)
```

---


### getLocation

```
public java.lang.String getLocation()
```

---


### setLocation

```
public void setLocation(java.lang.String location)
```

---


### getName

```
public java.lang.String getName()
```

---


### setName

```
public void setName(java.lang.String name)
```

---


### getOldFileName

```
public java.lang.String getOldFileName()
```

---


### setOldFileName

```
public void setOldFileName(java.lang.String oldFileName)
```


---


|  |  |  |
| --- | --- | --- |
| **Overview** \| **Package** \| **Class** \| **Use** \| **Tree** \| **Deprecated** \| **Index** \| **Help** | |  |
| **PREV CLASS**   **NEXT CLASS** | **FRAMES**    **NO FRAMES**     **All Classes** |  |
| SUMMARY: NESTED \| FIELD \| CONSTR \| METHOD | DETAIL: FIELD \| CONSTR \| METHOD |  |


---
